# Antibody H3 Structure Prediction

**DOI:** 10.1016/j.csbj.2017.01.010

**Published:** 2017-02-01

**Authors:** C. Marks, C.M. Deane

**Affiliations:** Department of Statistics, University of Oxford, 24-29 St Giles', Oxford OX1 3LB, United Kingdom

**Keywords:** Antibodies, H3, Loop modelling, Protein structure prediction

## Abstract

Antibodies are proteins of the immune system that are able to bind to a huge variety of different substances, making them attractive candidates for therapeutic applications. Antibody structures have the potential to be useful during drug development, allowing the implementation of rational design procedures. The most challenging part of the antibody structure to experimentally determine or model is the H3 loop, which in addition is often the most important region in an antibody's binding site. This review summarises the approaches used so far in the pursuit of accurate computational H3 structure prediction.

## 1 Introduction

Antibodies are proteins that bind to foreign objects that find their way into an organism, preventing them from causing harm and marking them for removal. A huge number of different antibodies can be produced – estimates vary, but it is thought that humans have the potential to of produce up to 10^13^ different antibodies [1] – making them capable of binding to a huge range of substances, ranging from proteins on the cell surface of bacteria to non-biological small molecules [2]. The substance that an antibody binds to is known as an antigen, and the specific region of the antigen to which the antibody binds is called the epitope. Mature antibodies bind with high affinity and are specific, meaning that they bind to other epitopes only very weakly, or not at all [3].

The ability of antibodies to bind with high affinity and specificity to their targets means that they are good candidates for therapeutic and diagnostic applications. Since the first antibody treatment, muromonab, was approved in 1986 for the prevention of transplant rejection, the market has grown rapidly [4]. By 2012, antibody therapies accounted for over a third of the total sales in the biopharmaceutical sector in the US, and they are currently the biggest-selling class of biopharmaceuticals [5].

Although molecules from biological sources tend to be larger, more complex and far more difficult to characterise than traditional small molecule drugs [6], they are promising as therapeutic agents [7]. Antibodies have been used for many disease areas: some currently on the market include infliximab (Remicade) and adalimumab (Humira) for the treatment of rheumatoid arthritis; trastuzumab (Herceptin) and bevacizumab (Avastin) for cancer; and alemtuzumab (Lemtrada) for multiple sclerosis [8].

Knowledge of an antibody's structure is extremely useful when developing a novel therapeutic, allowing it to be engineered more rationally. This knowledge can be used to increase binding affinity by guiding residues to be mutated, through the use of computational techniques such as binding affinity prediction [e.g. Ref. 9], epitope and paratope prediction [10,11], stability measurements [e.g. Ref. 12], and docking [e.g. Ref. 13]. Computational tools have already been used successfully to increase the binding affinity of antibodies [e.g. Refs. [14], [15], [16], [17], [18]. However, since experimental structure determination is time-consuming and expensive, the ability to computationally build accurate models of antibody structures (in particular their antigen-binding sites) from their sequences is highly desirable. This has become even more important as next-generation sequencing (NGS) data for antibodies has become available [1, 19].

## 2 Antibody Structure and the H3 Loop

Antibodies vary from large, multi-chain and multi-domain complexes, like those found in humans, to small, single domain molecules, such as nanobodies [20]. However, binding always occurs in a similar fashion, through interactions between the antigen and a number of loops on the antibody called complementarity determining regions (or CDRs). In standard mammalian antibodies, there are six of these loops; three on the heavy chain and three on the light chain (labelled L1, L2, L3 and H1, H2, H3 respectively). In contrast, for camelid antibodies, which lack a light chain, there are only three. The CDRs are the most variable parts of the whole antibody structure, and they govern the majority of the antigen-binding properties of an antibody.

The conformational diversity of five of the six CDRs (L1, L2, L3, H1 and H2) is thought to be limited. For these CDRs, only a small number of different shapes have been observed, forming a set of discrete conformational classes known as canonical structures [21]. Since its proposal in 1987 [21], the idea has been reinvestigated many times as the number of known antibody structures has increased [e.g. Refs. [22], [23], [24]]. These studies have led to the identification of particular amino acids at certain positions that are thought to be structure-determining; the canonical class of a CDR of unknown structure can therefore be predicted from its sequence with high accuracy. The least diverse CDR is L2, with around 99% of known structures belonging to the same class [24].

**Fig. 1:**
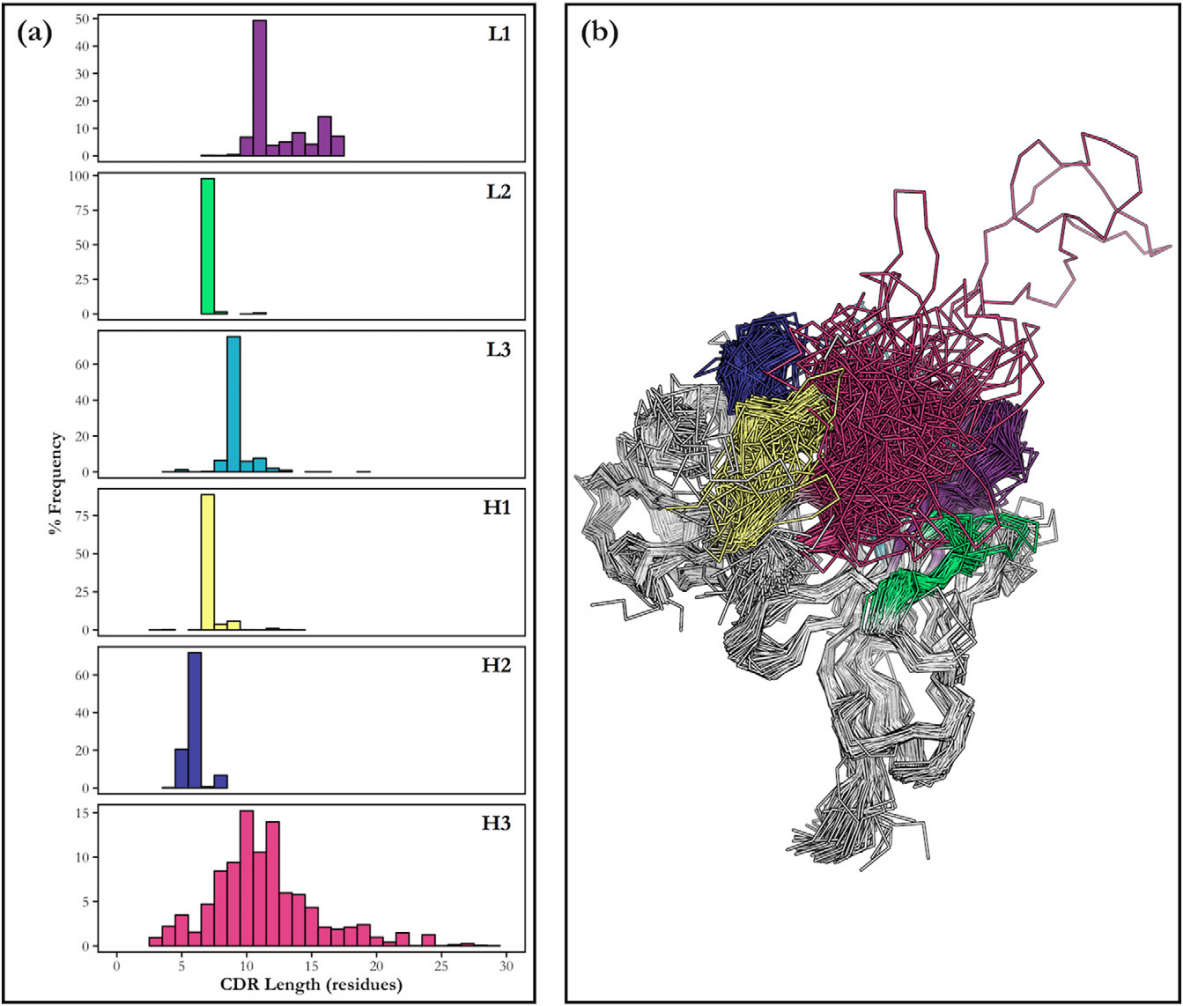
(a) The frequency of observed loop lengths for the six CDRs. Data shown is calculated from all structures in SAbDab [25] . The H3 loop displays greater diversity in length than the canonical CDRs. (b) The structures of a set of antibodies with up to 80% sequence identity and a resolution of up to 3 Å, as downloaded from SAbDab [25] . Framework regions are shown in grey, while the CDRs are coloured (L1 — purple, L2 — green, L3 — blue, H1 — yellow, H2 — dark blue, H3 — pink). H3 loops display more conformational diversity than the other parts of the antibody. (For interpretation of the references to colour in this figure legend, the reader is referred to the web version of this article.)

Unlike the other five CDRs, the H3 loop has not been classified into canonical forms; a huge range of structures have been observed (Fig. 1). This is due to how antibody sequences are encoded in the genome. The complete nucleotide sequence coding for an antibody heavy chain is created by combining gene segments from different locations (this is known as V(D)J recombination, after the ‘variable’, ‘diversity’, and ‘joining’ segments). The DNA encoding the H3 loop is found at the join between the V, D and J gene segments, which, with the addition of a process called junctional diversification, leads to a huge range of possible sequences. H3 loops vary widely in length: most are between 3 and 20 residues but they are occasionally far longer (Fig. 1). Bovine antibodies, for example, have H3s that are 50 or even 60 residues in length [26]. For comparison, the canonical CDRs each have a most 8 different lengths, and are normally far shorter - the longest canonical form is 17 residues long, but there are few examples of these five loops with lengths over 15 [23].

**Fig. 2:**
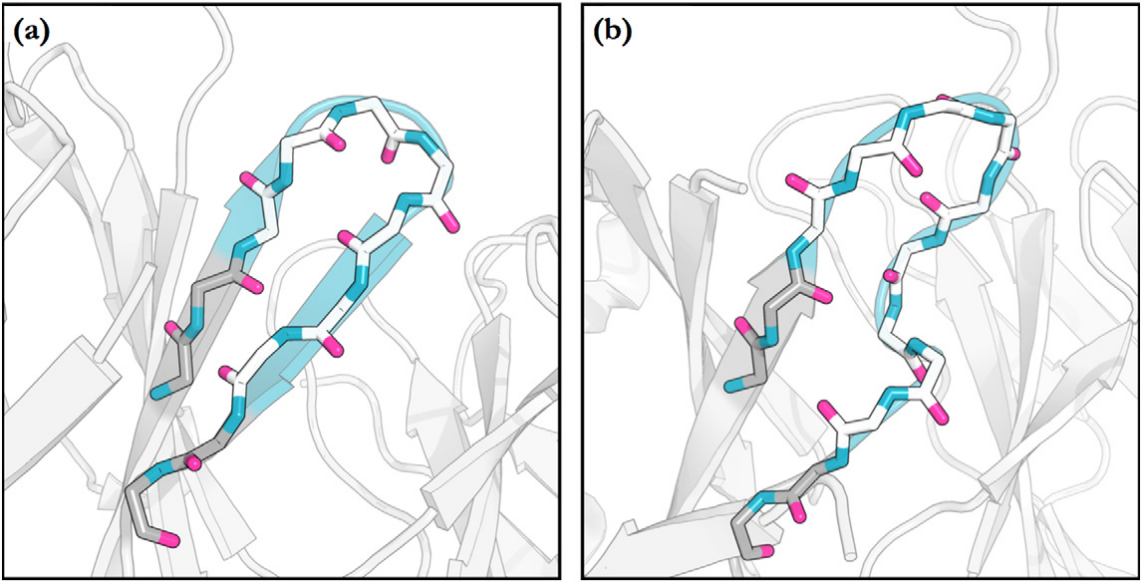
The ‘torso’ region of the H3 loop has been observed in two conformations: extended (a) and kinked (b). The backbone of the H3 loop and anchor residues are shown in stick representation, with carbons in white for the H3 loop and grey for the anchor residues. The majority of H3 structures are kinked.

The ‘torso’ of H3 loops (the residues nearest to the anchors) has been observed to adopt one of two conformations, labelled kinked (or bulged) or extended (or non-bulged — see Fig. 2). The majority of H3 loops are kinked [23], [27]. Proposals have been made about why this is the case, such as the interaction of a basic residue in theC-anchor with an asparagine located within the loop, which have led to the development of rules that aim to predict which conformation will be adopted [27], [28]. However, as more antibody structures have become available, these guidelines have been revisited and found to fail in some cases [23].

It is the H3 loop that is thought to contribute the most to an antibody's antigen-binding properties [8]. It is located in the centre of the binding site, and normally forms the most contacts with the antigen [29], [30]. It has also been shown to have the greatest effect on the energetics of binding [31], and to be the part of the antibody structure that changes the most upon binding [32]. Due to its location, the H3 loop contributes largely to the topography of the binding site — long H3s can create finger-like protrusions, and short H3s create cavities in the antibody surface, with a specific shape that only allows certain antigens with smaller or protruding epitopes to bind [33]. Knowledge of H3 structures is therefore extremely useful, enabling predictions to be made about antibody binding properties [e.g. Refs. [8], [11], [14], [15], [16], [17], [18], [34].

## 3 H3 Modelling Approaches

H3 structure prediction is a specific case of protein loop modelling. The starting point of a loop modelling problem is a series of missing residues in a protein structure, where the sequence of the missing segment is known but the three-dimensional structure of those residues is not. The protein structure used as input may be an experimentally-determined one, or a model. Predicting the structure of the loop requires three main steps: decoy generation, filtering, and ranking. In a similar way to the prediction of whole protein structures, where methods can be template-based or template-free, loop modelling algorithms can be divided into two categories depending on whether known structures are used in the decoy generation step. These categories are known as knowledge-based and *ab initio*.

### 3.1 Decoy Generation

**Fig. 3:**
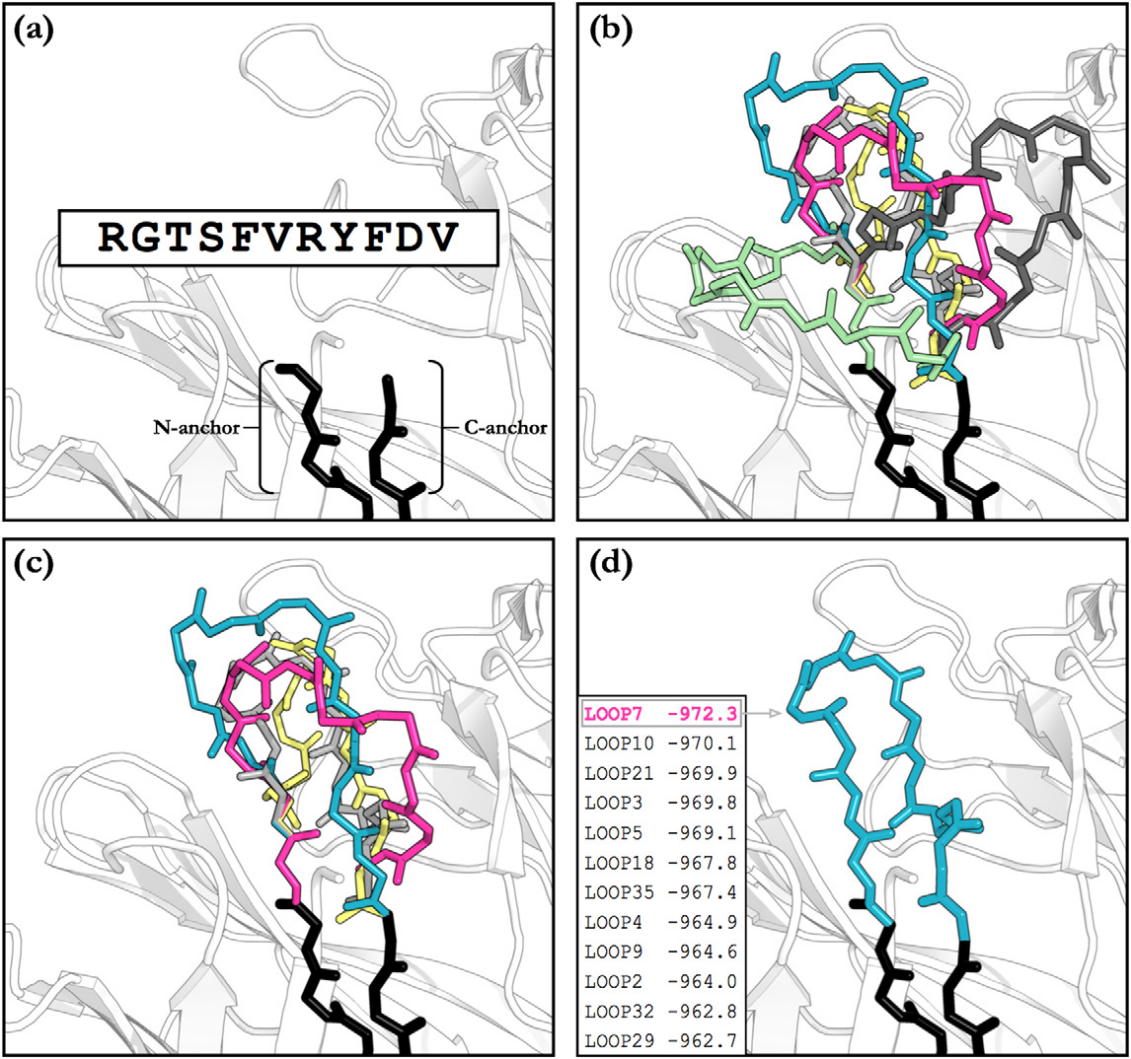
The main steps in an H3 loop modelling algorithm. (a) The inputs to the algorithm are an antibody structure with a missing loop, and the sequence of that loop. (b) Decoy generation. (c) Filtering of structures that are physically impossible, e.g. ones that clash with the rest of the structure. (d) Ranking and selection of the final prediction.

When predicting any loop structure, the first step is to generate a set of candidate conformations, or decoys, that connect the residues on either side of the gap in the protein structure. These neighbouring residues are termed the anchors; specifically the N-anchor for the one closest to the N-terminus of the sequence and C-anchor for the one nearest the C-terminus (Fig. 3).

As previously stated, methods for predicting protein loop structures are divided into two categories, knowledge-based or *ab initio*, depending on how they generate possible conformations (decoys). Knowledge-based methods rely upon databases of previously observed protein structure fragments. Structures are selected according to certain criteria such as fragment length (i.e. they must be the same length as the target loop), fragment-target sequence similarity and how closely the anchor geometry of the fragment matches that of the target loop. Methods of this type are fast, and can be very accurate when the structure of the target loop is similar to one previously observed [35]. However, there is not currently enough structural data to cover the conformational space, especially for long loops [36]. When a similar loop structure has not been observed previously, knowledge-based methods either give poor predictions or fail to return a prediction at all. Examples of this type of algorithm include FREAD [35], [37], SuperLooper [38], LoopWeaver [39] and LoopIng [40].

*Ab initio* methods do not rely on previously observed structures; instead, decoys are produced computationally. *Ab initio* methods work by exploring the possible conformational space, for example by randomly sampling the *ϕ* and *ψ* dihedral angles of the loop. While this allows novel structures to be generated, like knowledge-based methods *ab initio* algorithms have their limitations: they are computationally expensive, since many decoys must be generated to sample the conformational space sufficiently; and their prediction accuracy decreases with loop length (as the number of degrees of freedom increases). *Ab initio* algorithms include PLOP [41], Modeller [42], Loopy [43], LoopBuilder [44], LEAP [45], and the loop modelling routine within Rosetta [46].

The idea of a hybrid loop modelling algorithm, combining knowledge-based and *ab initio* approaches, has been explored. CODA [37] generates decoys using a knowledge-based method and an *ab initio* method separately, then combines the two decoy sets and makes a consensus prediction. Martin et al. [47], Whitelegg and Rees [48], and Fasnacht et al. [49] have used similar approaches, and applied it to modelling H3 loops — initial conformations are selected from a database of structures, and the middle section is then remodelled using *ab initio* techniques. An alternative approach using Rosetta is described by Rohl et al. [50] — this used a Monte Carlo-based fragment assembly method, in conjunction with a minimisation protocol.

Depending on how the loops are built, the continuity of the protein backbone may need to be enforced through the implementation of a closure algorithm. Alternatively, a minimisation step may be introduced, where the energy function has a term that penalises an ‘open’ loop. Three types of loop closure algorithm exist: analytical, iterative or build-up. Analytical methods calculate the values of particular degrees of freedom that are required to produce a continuous backbone (for example, *ϕ*/*ψ* angles). This approach was first introduced by Go and Scheraga [51] — they showed that the *ϕ*/*ψ* values necessary to close a loop can be solved mathematically for up to six angles. This approach is used to maintain loop closure in the loop modelling routine within Rosetta, in the algorithm called kinematic closure or KIC [52]. Similar algorithms are used in robotics, to move multi-jointed ‘arms' to specific locations in space [36].

Iterative methods normally start with an open conformation, and gradually enforce its closure through a series of steps. A key example of this type is cyclic coordinate descent, or CCD [53] — starting at one end of the loop, each *ϕ* or *ψ* angle is altered so that the distance between the free end of the loop and the fixed anchor is minimised. This continues iteratively, until the distance between the two ends is low enough to consider the loop closed. The change in angle required is calculated analytically; CCD can therefore be thought of as both an analytical and an iterative method.

Build-up methods attempt to guide loop building such that a closed loop conformation is automatically generated. RAPPER, for example, builds loops starting from the N-anchor, and places restraints on each C*α* atom added to the structure, limiting the distance they are allowed to be from the C-anchor [54]. Loop closure is enforced by making the restriction gradually tighter as more residues are added.

### 3.2 Filtering

Some of the decoys generated will not be physically possible. For example, *ϕ*/*ψ* angles of the structure may be in the disallowed regions of the Ramachandran plot, or atoms may be too close together. A filtering step is therefore required to remove these structures. This step may be combined with the other parts of the loop modelling process; for example some algorithms combine it with decoy generation itself. The Direct Tweak loop closure method, for example, enforces a continuous backbone while monitoring the loop for clashes [43].

### 3.3 Ranking

Once all decoys have been generated, a ranking system is needed to select a final prediction; i.e. the one that is predicted to be closest to the true structure of the target (the native structure). This is a vital step; even if decoys close to the native structure have been generated at a previous stage, an ineffective ranking system means that the structure chosen as the final prediction will be inaccurate.

For knowledge-based methods, the ranking system may use properties of the decoy/fragment structure — for example the similarity between the target sequence and the decoy sequence, or between the geometry of the decoy anchors and the anchors of the target. FREAD, for example, ranks the fragments selected from a database by the root mean square deviation (RMSD) between the atomic positions of the target and fragment anchor residues [35], [37].

More commonly, especially for *ab initio* methods, an energy function is used to predict which structures are lower in energy and therefore more likely to be near-native. There are two main types of energy function: physics-based force fields and statistical potentials [55]. Force-fields are equations with separate terms for the contribution of different properties to the energetics of a system. These include bonded interactions, such as bond lengths, bond angles, and dihedral angles; and non-bonded interactions, like electrostatics and van der Waals' forces [56]. Further terms must also be added that consider the effect of solvation; this can be done using either an implicit model, which treats the solvent as a continuous medium (e.g. the Generalized Born model [57]), or the water can be treated explicitly, meaning that individual water molecules are added to the system (for example the TIP4P model [58]). The terms are parameterised using empirical evidence. Some examples of force fields are AMBER [59], CHARMM [60] and OPLS [61].

Statistical (or knowledge-based) potentials use pre-observed structures to infer the relative energy of a protein, based on the assumption that the distributions of particular structural features seen in nature reflect energetics [36], [55]. For example, the carbon to oxygen bond in the carbonyl of the protein backbone is regularly observed in experimentally-determined structures to have a length of 1.23  Å [62] — a decoy with a C–O length of around this value is therefore likely to be more energetically stable than one with a C–O distance of 2  Å. Statistical potentials are attractive because the protein energetics do not necessarily need to be deciphered — these functions incorporate unknown or poorly-understood interaction terms into their calculation without having to explicitly include them [55]. In addition, they are often faster to run than force field calculations, automatically include solvation and the potential can be smoother — small changes in conformation do not lead to huge differences in energy. Examples of statistical potentials include DFIRE [63], DOPE [64], GOAP [65] and SOAP-Loop [66].

## 4 H3 Structure Prediction: Algorithms and Accuracy

Due to its structural diversity, structure prediction of the H3 loop is challenging. However, it is possibly the most important part of the structure to model correctly, since it is thought to be mainly responsible for the antigen-binding properties of an antibody [8]. While some algorithms exist that do not treat it any differently to the other CDRs (e.g. ABGEN [67]), this is not usual and a special approach is normally implemented, using a knowledge-based or *ab initio* approach, or some combination of the two. Some algorithms have tried to use the presence of a kinked or extended conformation to guide H3 loop modelling, by using a series of rules to predict which conformation is adopted from sequence. This information can then be used to either pre-filter a database of solved structures in the case of knowledge-based methods [49], or limit the conformational search of an *ab initio* algorithm [68], [69], [70].

The current accuracy of antibody structure prediction is monitored through a CASP-style [71] blind prediction test called the Antibody Modelling Assessment (or AMA). The first AMA was conducted in 2011 [72] — participants were given the sequences of several unpublished, high-resolution antibody structures and asked to model them. More recently, the results of the second assessment (AMA-II) were published [73]. AMA-II featured two rounds: the first entailed modelling the entire variable region (or Fv) from its sequence; in the second, the accuracy of H3 structure prediction was tested in isolation by giving the participants the native structures of the Fv regions with the H3 loop residues missing. Any errors introduced into the H3 model caused by inaccuracies in the framework structure are therefore eliminated, giving an impression of the current accuracy of H3 prediction. Each group was required to submit five predictions for each of ten H3 targets, with loop lengths ranging from 8 to 14 residues.

The group that achieved the best results was Schrödinger, using the commercial Prime software. The loop modelling algorithm is freely available under the name PLOP [41]. For eight of the ten targets, Prime produced the most accurate model. However, as is the case for all the groups, once all five of the predictions are taken into account instead of only the best, average RMSDs become far worse. There are several possible reasons for this: the set of loop models generated may only contain a couple of good models; or the ranking system used to select good models is inadequate. Alternatively, the five predictions for each target may have been purposefully chosen so that they cover a larger conformational space between them, preventing the submission of five very similar but incorrect models. This indicates that the ranking method used cannot consistently choose the best conformations.

The results obtained during AMA-II, as well as some other H3 prediction studies, are shown in Table 1. Reasonable accuracies are currently being achieved for short H3 loops (up to around 9 residues), but predictions become far worse for loops beyond that length. There is an appreciable difference in accuracies achieved modelling H3 loops compared to the other CDRs. For example, the knowledge-based method FREAD has been shown to produce sub-ångström predictions for the five canonical CDRs (0.81  Å, 0.42  Å, 0.96  Å, 0.98 Å and 0.88  Å for L1–H2), while the average accuracy for H3 loops is 2.25  Å [74]. RosettaAntibody also produces sub-ångström predictions for the other CDR loops (0.78  Å, 0.54  Å, 0.81  Å, 0.84  Å and 0.93  Å), while the accuracy of H3 prediction ranges between 1.6  Å and 6.0  Å depending on length.

The following sections provide details of the algorithms whose accuracy is reported in Table 1. Although the algorithms described are all H3-specific, or have been used to model H3 loops, they give an overview of the methodologies used for loop modelling in general.

### 4.1 ABGEN

ABGEN (the AntiBody structure GENeration algorithm) is an antibody modelling tool published by Mandal et al. [67]. There are two parts to the algorithm: ABalign, which selects a template structure for each part of the structure (i.e. framework and CDRs) by sequence similarity; and ABbuild, which is responsible for generating the three-dimensional structure. The CDRs are modelled using a knowledge-based approach, and the H3 loop is not treated any differently — candidate templates are found from known antibody structures and selected based on sequence and length. If no loop exists of the same length, then the closest is selected. The loops are grafted onto the framework structure by superimposing the anchor residues (the two residues on either side of the loop). Residue mis-matches between the template and target are then dealt with by replacing the sidechains. Clashes are avoided by iteratively changing the sidechain torsion angles. Prediction of the whole antibody structure is reported to take around 5 min [67].

### 4.2 Accelrys Tools

Accelrys is a software company that has produced an antibody prediction tool for commercial use (accelrys.com/products/collaborative-science/biovia-discovery-studio). Its performance was evaluated during AMA-II [49]. Three different methods are used to predict the H3 loop:

1. a purely knowledge-based approach — templates are selected from a database like they are for the other CDRs, except the kinked/extended conformation is also considered.
2. like method 1, with additional remodelling of the most variable part of the loop using an *ab initio* approach. The section of the loop to be remodelled is chosen by eye.
3. like method 2, but with additional sidechain refinement before the *ab initio* modelling — the sidechains of the H3 loop are mutated to alanine while those of the rest of the structure are refined.

During the second round of AMA-II (H3 modelling onto the native antibody structure), method 2 was used. The final decoy selection is carried out based on clustering — all conformations are grouped by structural similarity, and the clusters are ranked according to the energy of its members (calculated using a physics-based energy function). The lowest energy model from the top-ranked cluster is given as the final prediction. On average, the algorithm takes 30 min to produce a prediction [49].

### 4.3 CCG (MOE)

The protocol used by the (Chemical Computing Group), or CCG (part of the commercial MOE software, see chemcomp.com/ MOE-Molecular_Operating_Environment.htm) is a knowledge-based algorithm, used in conjunction with molecular dynamics [75]. Its performance was evaluated during AMA-II. Known H3 structures are scored based on backbone topology, bond lengths and angles, probability of *ϕ*/*ψ* angles, crystallographic occupancies, and temperature factors. After clustering, the member of the cluster with the highest score is put into a database. This database is enriched by running molecular dynamics (MD) simulations on these structures. Possible structures are selected from the database depending on anchor RMSD, using a tight cutoff of 0.25  Å, and a final prediction is made using a score that takes into account H3-specific properties, such as surface-accessible surface area, *ϕ*/*ψ* angles, and the interaction of the loop to the rest of the Fv. During the AMA-II, the production of each full antibody model took around 30 min [75].

### 4.4 FREAD and ConFREAD

FREAD is a knowledge-based method that selects possible loop conformations from a database of experimentally determined protein fragments [35], [37]. It is freely available to use online (opig.stats.ox.ac.uk/webapps/fread/php). It is used as the CDR structure prediction method within the ABodyBuilder antibody modelling software [81], [82]. Loops are initially selected as potential predictions according to the separation of their anchor residues compared to that of the target and their sequence similarity to the target loop. The fragments are filtered depending on whether their insertion into the protein structure would cause clashes. These fragments (assuming some suitable fragments are found) are then ranked by the RMSD between the anchor residues of the fragment and those of the target loop; the loop with the lowest RMSD is assumed to have the most similar structure to the target and is hence returned as the final prediction. If no suitable fragments are found, however, FREAD does not produce a prediction. The computational time required varies with the size of the database being searched, but is normally around 1–2 min.

Research into improving FREAD's ability to predict H3 loops led to a new version with an additional filter that considers the contact profiles of the fragments within the database [74]. Each residue of each fragment in the database is annotated with a number depending on the contacts it forms in its native environment: 0 for no contacts; 1 for external contacts (those to another chain); 2 for internal contacts (formed with other residues on the same chain); and 3 for both internal and external contacts. The actual contact profile of the fragment is then compared to its profile when inserted into the target structure — only fragments with matching pairs are retained. The final prediction was chosen in the same way as in the original FREAD algorithm. While this led to an increase in prediction accuracy (from 2.25  Å to 1.23  Å), coverage (the proportion of targets for which the algorithm could produce a prediction) was significantly lower (reduced to 70% from 100%).

### 4.5 H3Loopred

H3Loopred [76] is a knowledge-based method that uses machine learning to predict which of a set of H3 structures is closest to the desired target structure. The software is available for download from biocomputing.it/H3Loopred. A Random Forest model was developed that uses several features to predict the similarity of a known loop structure to the structure of the target, using a measure called the TM score [83]. The features used are a mixture of general and H3-specific properties: loop sequence, the canonical classes and lengths of the other CDRs in the antibody, source organism, germline family, and the similarity scores for each residue and the whole loop. If the structure from the database that is predicted to be the best has a predicted TM score of less than 0.5, then this loop is returned as the final prediction. Otherwise, the top 50 templates are ranked using a score that considers contacts. The average computation time required is 5 min per target [76].

### 4.6 Kotai Antibody Builder

Kotai Antibody Builder is a simplified and automatic version of the software used in AMA-II by the joint Osaka University Astellas (JOA) team [77], [84]. An online server is available at kotaiab.org. In the second round of AMA-II, H3 decoys were generated using a combination of a knowledge-based approach with molecular dynamics simulations. Spanner (the knowledge-based algorithm) selects fragments from a database, filtering them using sequence similarity, secondary structure similarity, a clash score, the geometry of the anchor residues, and the predicted kinked/extended conformation of the loop. Minimisation of these structures is carried out using the OSCAR-loop energy function [45], and the top 20 structures are used as initial conformations for a series of MD simulations. Snapshots of the simulations are then grouped into five clusters, with the final set of predictions including one structure from each.

### 4.7 BioLuminate and Prime

BioLuminate and Prime are software packages produced by the Schrödinger company (schrodinger.com/products/bioluminate); their performance was evaluated in the AMA-II [79]. Prime is the commercial version of the loop modelling algorithm PLOP (the Protein Local Optimisation Program, [41]). BioLuminate models antibodies using homology; CDRs (including H3) are modelled by selecting templates from a database. Prime, on the other hand, is an *ab initio* algorithm. For stage 1 of AMA-II, where H3 predictions were made onto model frameworks, the three submitted models were generated in different ways: a straightforward template selection based on sequence similarity; template selection after clustering known H3 structures, taking the structure with the highest sequence similarity from the largest cluster; and *ab initio* prediction using Prime. In the second AMA-II round, the *ab initio* approach was used exclusively, but the target loop was extended by one residue on each side to make the terminal residues flexible.

Prime uses a hierarchical approach to model protein loops: a ‘full’ prediction job is made up of many ‘standard’ jobs. Like many other *ab initio* methods, in a standard job loops are built by choosing random *ϕ*/*ψ* angles from Ramachandran distributions. However, instead of building loops by adding all residues onto one of the anchors with subsequent closure, they are built in two segments — half onto the N-anchor and half onto the C-anchor. Many structures are created for each half of the loop. All N-anchor segments are then compared against all C-anchor segments to find pairs that meet in the centre, thereby forming a complete loop structure. Decoys that have unrealistic dihedral angles or clash with the rest of the protein are filtered out, and all remaining loop structures are then clustered, and the representative structures (those from the centre of each cluster) undergo energy minimisation.

A full prediction job is then made up of a series of standard jobs, with the conformational search space becoming narrower at each stage. By using the predicted best structures generated during previous stages to constrain the conformational search, the algorithm is guided towards creating structures of low energy. The final step is the ranking of all loop structures that were generated from all steps, according to their calculated energy; the loop with the lowest energy is returned as the final prediction.

### 4.8 RosettaAntibody

RosettaAntibody, which was one of the algorithms used during AMA-II [68], models the H3 loop using an *ab initio* approach. Loop modelling in the Rosetta protein modelling software (available from rosettacommons.org/software) is carried out using a kinematic closure protocol, made up of ‘KIC moves' [46], [52]. Prediction begins with the generation of a random loop structure. During a KIC move, three C*α* atoms of the loop segment are chosen as ‘pivots', leaving the remaining C*α* atoms as ‘non-pivots'. Dihedral angles of the non-pivots are sampled from Ramachandran distributions. The dihedral angle changes of the pivots required to maintain loop closure are then calculated analytically. The full protocol, which includes sidechain optimisation and backbone energy minimisation, involves carrying out KIC moves iteratively, with different pivot atoms each time. The lowest scoring model, according to the statistical Rosetta scoring function, is reported as the final loop prediction.

‘Next-generation KIC’ is an new version of this algorithm [46]. This improved protocol includes the sampling of *ω* dihedral angles, neighbour-dependent *ϕ*/*ψ* sampling, and annealing (the weights of certain terms in the Rosetta energy function are slowly increased so that they have less of an effect initially, allowing the barriers between energy minima to be overcome).

For the first stage of AMA-II, models were generated using next-generation KIC without using any neighbour dependence during *ϕ*/*ψ* sampling. In stage 2, both this approach and ‘legacy KIC’ were used (the original version published by Mandell et al. [52]), and for those targets predicted to have a kinked conformation, constraints were added to enforce it. A more recent paper has explored this idea further, and has shown that the addition of the kink constraint improves sampling and therefore overall prediction accuracy [70].

### 4.9 SmrtMolAntibody

SmrtMolAntibody, the commercial antibody modelling software developed by Macromoltek (see macromoltek.com), was also tested during AMA-II [69]. An *ab initio* approach is used to model the H3 loop. Firstly, the first and final three residues of the loop are modelled according to their predicted kinked/extended conformation. The remaining residues are then added as dimers, where the *ϕ*/*ψ* angles of the two residues have been observed together in nature. After all decoys are generated, the structures are filtered, by checking each trimer for non-physical neighbouring dihedral angles, and finally ranked using a statistical potential. The reported time required to produce a full antibody model is 30 min [69].

### 4.10 WAM

WAM (Web Antibody Modelling) uses different approaches to model H3 loops depending on their length [48]. If the loop is shorter than eight residues, then a traditional knowledge-based algorithm is used. Specific databases are used depending on whether the loop is predicted to have a kinked or extended conformation. For loops of eight residues or more, the database search is followed by the remodelling of the middle five residues of the loop using an *ab initio* method, CONGEN (Conformation Generator) [85]. CONGEN produces decoys by calculating *ϕ*/*ψ* angles that form a closed structure, using the work of Go and Scheraga [51]. The decoys undergo minimisation, and are clustered to remove duplicate conformations. The final prediction is selected from the pool of decoys using a score that considers surface accessibility, the RMSD of the decoy to known kinked H3 structures, and the calculated energy (using a physics-based energy function).

### 4.11 Sphinx

Sphinx is a recently-developed method that integrates knowledge-based and *ab initio* approaches [80]. An H3-specific version is freely available for use at opig.stats.ox.ac.uk/webapps/sabdab-sabpred/SphinxH3.php. The algorithm starts with a database search; loop fragments that are shorter than the target loop are extracted based on their sequence similarity to the target. The structural information within a fragment is then used to build decoys according to the alignment of its sequence to the target's — i.e. for residues in the target loop that are matched with a fragment residue in the alignment, the residue is modelled using the bond lengths, angles and dihedral angles of the matching fragment residue. If a target residue is not matched with a fragment residue, then the necessary information is drawn at random from relevant distributions, as in a straightforward *ab initio* algorithm. Loop closure is enforced using the CCD algorithm [53]. Each selected fragment is used to generate 100 decoys, leading to a large set of possible conformations. Using a knowledge-based energy function, the number is reduced to 500, and these are subsequently minimised using Rosetta [52] and ranked using the statistical potential SOAP-Loop [66].

## 5 Conclusions

While the accuracy of H3 structure prediction has improved in recent years, as evidenced by the results of the two Antibody Modelling Assessments [72], [73], the modelling of H3 loops remains the biggest challenge in producing accurate and useful antibody models. There remains a marked difference between the accuracy of H3 prediction compared to that of the canonical CDRs: these five loops are regularly predicted with sub-ångström accuracy while H3 prediction accuracy is much more variable, typically with an RMSD of between 1.5 and 3  Å, but often worse, in particular in the non-native environment. Since overall, the aim of this research area is to produce accurate models that can assist in the rational design of antibody therapeutics, the key results are those reported for H3 prediction in the non-native environment — it is obvious from results reported in the literature so far that improvements must still be made to enable the production of useful antibody models. An aspect of H3 prediction that is particularly challenging, identified as difficult by the organisers of AMA-II [73], is the accurate scoring of decoy structures — even if good conformations are made during decoy generation, it is often the case these decoys are not selected as final predictions due to poor ranking. Further developments in this area, along with continuing improvements to the accuracy of framework and canonical CDR modelling, would be of great benefit.

The type of algorithms that were used in the second Antibody Modelling Assessment, considered to be the state-of-the-art, imply that there is a general movement away from purely knowledge-based loop modelling approaches when it comes to H3 structure prediction. Only one of the six algorithms examined (CCG's MOE software) could be classified as a knowledge-based approach, and this, along with the results shown in Table 1, are an indication that H3 structures (especially those that are long) are too diverse to be consistently modelled accurately at the current time using only previously-observed structures. By using more restrictive selection parameters, the performance of a knowledge-based algorithm can be improved (as in the case of ConFREAD), however coverage must be compromised and some other method must be used to model loops for which no close structural match can be found in the PDB. Using an *ab initio* method alone, on the other hand, means that any useful structural information that is available is ignored. The next logical step, then, is a hybrid method which takes advantage of both approaches. The development of such methods has already begun, with the prediction software of Accelrys Tools [49] and KotaiAntibodyBuilder [77] being assessed during AMA-II, the latest Rosetta algorithm, which uses knowledge of H3 structures to constrain the torso of the loop into a kinked conformation [70], and the more recent Sphinx algorithm [80]. Further investigations into how the two approaches may be integrated should lead to more accurate, and hence more useful, antibody models.

## Figures and Tables

**Table 1:** Reported accuracies for H3 prediction achieved by some loop modelling algorithms. Values given are average global RMSDs for the number/length of loops indicated. Some target sets are the same, indicated by a * or † symbol. RMSDs quoted for the AMA-II target set (denoted by *) are carbonyl RMSDs, i.e. calculated over the C and O atoms of the backbone only. Unless otherwise stated, predictions were made in the crystal environment (i.e. using the antibody structure determined experimentally).

Algorithm	Type	Key results	Ref.
ABGEN	KB	Model environment:	[67]
		2.3 Å (15 loops, lengths 5–17, model environment)	
		(1.9 Å for up to 10 residues, 3.0 Å for over 10 residues)	
Accelrys Tools	KB+AI	Model environment:	[49]
		Best of top 3 = 3.14 Å; average of top 3 = 3.88 Å (11 loops, lengths 8–14)*	
		Crystal environment:	
		Best of top 5 = 1.86 Å; average of top 5 = 2.89 Å (10 loops, lengths 8–14)*	
CCG (MOE)	KB	Model environment:	[75]
		Best of top 3 = 2.86 Å; average of top 3 = 3.69 Å (11 loops, lengths 8–14)*	
		Crystal environment:	
		Best of top 5 = 2.09 Å; average of top 5 = 3.08 Å (10 loops, lengths 8–14)*	
FREAD	KB	2.25 Å (97 loops, lengths 3–19, coverage = 100%)	[74]
ConFREAD	KB	1.23 Å (97 loops, lengths 3–19, coverage = 70%)	[74]
H3Loopred	KB	Model environment:	[76]
		1.3 Å (3 loops, lengths 4–6)^†^ 3.3 Å (10 loops, lengths 12–14)^†^	
		1.6 Å (22 loops, lengths 7–9)^†^ 7.1 Å (4 loops, lengths 17–22)^†^	
		1.8 Å (14 loops, lengths 10–11)^†^	
KotaiAntibody-builder	KB+AI	Model environment:	[77]
		Best of top 3 = 2.41 Å; average of top 3 = 3.02 Å (11 loops, lengths 8–14)*	
		Crystal environment:	
		Best of top 5 = 1.25 Å; average of top 5 = 2.43 Å (10 loops, lengths 8–14)*	
		0.18 Å (3 loops, lengths 4–6)^†^ 2.38 Å (10 loops, lengths 12–14)^†^	[78]
		0.70 Å (22 loops, lengths 7–9)^†^ 3.63 Å (4 loops, lengths 17–22)^†^	
		0.67 Å (14 loops, lengths 10–11)^†^	
Prime/PLOP	AI	Model environment:	[79]
		Best of top 3 = 2.74 Å; average of top 3 = 3.60 Å (11 loops, lengths 8–14)*	
		Crystal environment:	
		Best of top 5 = 1.12 Å; average of top 5 = 2.54 Å (10 loops, lengths 8–14)*	
		Crystal Environment:	
		1.6 Å (3 loops, lengths 4–6)^†^ 3.1 Å (10 loops, lengths 12–14)^†^	
		1.9 Å (22 loops, lengths 7–9)^†^ 6.0 Å (4 loops, lengths 17–22)^†^	
		2.4 Å (14 loops, lengths 10–11)^†^	
RosettaAntibody	AI	Model environment:	[78]
		1.4 Å (3 loops, lengths 4-6)^†^ 3.5 Å (10 loops, lengths 12–14)^†^	
		2.2 Å (22 loops, lengths 7-9)^†^ 7.6 Å (4 loops, lengths 17–22)^†^	
		2.9 Å (14 loops, lengths 10-11)^†^	
		Model environment:	[79]
		Best of top 3 = 2.66 Å; average of top 3 = 3.11 Å (11 loops, lengths 8–14)*	
		Crystal environment:	
		Best of top 5 = 1.97 Å; average of top 5 = 3.22 Å (10 loops, lengths 8–14)*	
		2.0 Å (44 kinked loops, lengths 9–19)	[70]
SmrtMolAntibody	AI	Model environment:	[69]
		Best of top 3 = 3.02 Å; average of top 3 = 3.71 Å (11 loops, lengths 8–14)*	
		Crystal environment:	
		Best of top 5 = 2.41 Å; average of top 5 = 3.08 Å (10 loops, lengths 8–14)*	
WAM	KB+AI	≤1.7 Å for 9 out of 11 loops under 10 residues	[48]
		1.3–2.7 Å for loops of 10 residues or more (8 loops, lengths 10–12)	
Sphinx	KB+AI	Crystal environment:	[80]
		Top prediction = 2.50 Å, best of top 5 = 1.52 Å (39 loops, lengths 4–22)	
		Best of top 5 = 1.41 Å; average of top 5 = 2.17 Å (10 loops, lengths 8–14)*	
		Model environment:	
		Top prediction = 3.26 Å, best of top 5 = 2.60 Å (39 loops, lengths 4–22)	
